# Supplementary Far-Red Light for Photosynthetic Active Radiation Differentially Influences the Photochemical Efficiency and Biomass Accumulation in Greenhouse-Grown Lettuce

**DOI:** 10.3390/plants13152169

**Published:** 2024-08-05

**Authors:** Haijie Dou, Xin Li, Zhixin Li, Jinxiu Song, Yanjie Yang, Zhengnan Yan

**Affiliations:** 1 College of Intelligent Science and Engineering, Beijing University of Agriculture, Beijing 102206, China; haijiedou@bua.edu.cn; 2 College of Horticulture, Qingdao Agricultural University, Qingdao 266109, China; 3 College of Agricultural Engineering, Jiangsu University, Zhenjiang 212013, China

**Keywords:** far-red, *Lactuca sativa*, leaf expansion, performance index

## Abstract

Adding far-red (FR, 700–800 nm) light to photosynthetic active radiation (400–700 nm) proved to be a possible approach to increasing plant biomass accumulation for lettuce production in indoor vertical farms with artificial lighting as a sole-source lighting. However, how FR light addition influences plant growth, development, and metabolic processes and the optimal value of FR photon flux density for greenhouse-grown lettuce under sunlight are still unclear. This work aims to quantify the value of supplementary FR light with different intensities on lettuce morphological and physiological characteristics in a greenhouse. Lettuce ‘Dasusheng’ (*Lactuca sativa* L.) was grown in a greenhouse under seven light treatments, including white plus red LEDs with FR photon flux density at 0, 10, 30, 50, 70, and 90 µmol m^−2^ s^−1^ (WR, WR + FR10, WR + FR30, WR + FR50, WR + FR70, and WR + FR90, respectively), and lettuce grown with sunlight only was marked as natural light (NL). FR light addition improved the electron transport flux per cross section and performance index (PI_abs_, PI_total_) and decreased the changes in relative variable fluorescence of lettuce leaves compared to plants under NL. Specifically, the PI_abs_ of lettuce leaves were 41%, 41%, 38%, 33%, 26%, and 25% lower under control than in plants under treatments WR + FR90, WR + FR70, WR + FR50, WR + FR30, WR + FR10, and WR, respectively. Leaf number, leaf area, and biomass accumulation of lettuce followed a quadratic function with increasing FR light intensity and were the highest under treatment WR + FR50. The shoot fresh weight and dry weight of lettuce were increased by 111% and 275%, respectively, under treatment WR + FR50 compared to NL. The contents of vitamin C, reducing sugar, total soluble sugar, and starch in lettuce showed a similar trend with biomass accumulation. In conclusion, with commonly used photosynthetic photon flux density (PPFD, 400–700 nm) around 200 μmol m^−2^ s^−1^, supplementary FR light intensity of 30~50 μmol m^−2^ s^−1^ was suggested to enhance the photochemistry efficiency, biomass accumulation, and carbohydrates’ contents in greenhouse-grown lettuce.

## 1. Introduction

In the past decade, a number of studies have focused on the influences of different light intensities or light spectra within photosynthetic active radiation (PAR, ranging from 400 nm to 700 nm) on plant growth in controlled environments [1,2,3,4]. On the contrary, far-red (FR, 700–800 nm) photons, which are out of PAR range, have received little attention and are often presumed to be less efficient for plant photosynthetic functioning compared to shorter wavelengths such as blue (B), green (G), or red (R) light [5,6,7]. However, in recent years, an increasing body of empirical evidence suggested that adding FR to PAR wavelengths improved lettuce (*Lactuca sativa* L.) biomass accumulation through stimulation of leaf expansion and increased the amount of light energy captured by leaves, as well as a better excitation between the two photosystems, photosystem I (PSI) and photosystem II (PSII) [6,7,8,9,10,11]. Zhen and van Iersel reported that the quantum yield of PSII (φPSII) and net photosynthetic rate (P_n_) in lettuce increased immediately by adding FR photons to combined R&B photons [R_76_G_1_B_2_3, subscript numbers indicate the photon percentage of R, G, and B wavelengths in the total photon flux density (TPFD, 400–780 nm), respectively] and white (W) photons (B_12_G_43_R_41_FR_4_), owing to a more balanced excitation of PSI and PSII [6]. Further, FR light is equally efficient at driving canopy photosynthesis when acting synergistically with PAR wavelengths among 14 plant species, while the magnitude of the effect was less at longer wavelengths (711, 723, and 746 nm) [7]. However, it was also reported that FR addition to combined R&B photons showed no influence on leaf P_n_ and decreased the plant’s photosynthetic capacity in the long term due to a lower chlorophyll and total nitrogen content, which reduced leaf absorbance [12,13]. Thus, it was postulated that the acclimation process of plant morphology triggered by FR addition plays a major role in improving the yield of indoor cultivated lettuce [9,13]. Legendre and van Iersel compared the efficiency of FR addition to PAR and PAR wavelengths related to light interception and biomass accumulation, and results indicated that FR light was 57% and 183% more effective at increasing the light interception received by the plant, as well as 92% and 162% more effective at increasing plant biomass at the early and late harvests, respectively, as compared to PAR wavelengths [10]. Accordingly, the enhanced crop production by FR addition, which was mainly caused by plant morphological responses, cannot be achieved by adding similar amounts of shorter wavelengths [10].

Although increasing the FR photon flux density may have beneficial effects on photosynthesis and biomass accumulation, this can happen at the expense of the contents of photosynthetic pigments. Lower levels of chlorophyll and carotenoids are often reported when plants are grown under FR addition [14,15]. Zou et al. reported that supplementary FR light during cultivation, especially under FR light during the day (FR photon flux density of 90 μmol m^−2^ s^−1^, PPFD of 200 μmol m^−2^ s^−1^ provided by a combination of red and blue light, photoperiod of 16 h), is associated with reduced nutraceutical quality at harvest and potentially shorter shelf-life, which was related to elevated O^2−^ content along with decreased activity of enzymatic (superoxide dismutase) and reduced levels of non-enzymatic (carotenoids, total phenolics, and flavonoids) antioxidants in lettuce plants [16]. The sugar content, on the other hand, is often increased when plants are grown under higher FR fractions, due to enhanced photochemistry efficiency and relative CO_2_ assimilation [8,16]. However, the regulatory mechanism still needs to be further studied.

Lettuce, the main ingredient in many salads and a popular sandwich topper, is one of the most widely consumed leafy greens around the world [17,18,19]. According to the Food and Agriculture Organization of the United Nations (FAO), China is the world’s largest lettuce producer, with an estimated 17.9 million metric tons to be produced in 2020, followed by the United States [20]. With a rising demand for lettuce production, production occurs year-round, especially with an increasing amount grown in controlled environments (e.g., greenhouses and indoor vertical farms, IVF). However, lettuce growth can be slow when the daily light integral (DLI, product of PPFD and photoperiod) is low, especially in greenhouses during early spring, late autumn, and winter time in northern latitudes [21]. Therefore, supplemental lighting, especially provided by light-emitting diodes (LEDs), is often used to increase the DLI for greenhouse lettuce production and obtain high-quality produce [21,22,23].

FR light is a critical signal that influences plant growth, development, and metabolic processes. To date, most studies have focused on the effect of FR light addition on lettuce morphological characteristics and photosynthetic indicators in a fully controlled environment (e.g., IVF) with artificial lighting as sole-source lighting [6,7,8,9,10,11,13,16], which may not be applicable to semi-closed agricultural facilities (e.g., greenhouses), one vital form for lettuce production. Further, the lighting environment in greenhouses is different from fully controlled facilities, in which few FR photons are provided by sunlight, as well as varying light intensity, photoperiods, and DLI, which may not be suitable for lettuce cultivation. Thus, this work aims to explore the influence of supplementary FR light with different intensities on lettuce morphological and physiological characteristics in greenhouses during low DLI periods. We hypothesized that in greenhouses with sunlight at a low DLI, supplementary PAR with FR photons would be effective in increasing lettuce growth, biomass accumulation, and sugar contents, and these morphological and growth responses should have a qualitative threshold.

## 2. Results

### 2.1. Chlorophyll: Fluorescence Parameters of Greenhouse-Grown Lettuce

**Figure 1:**
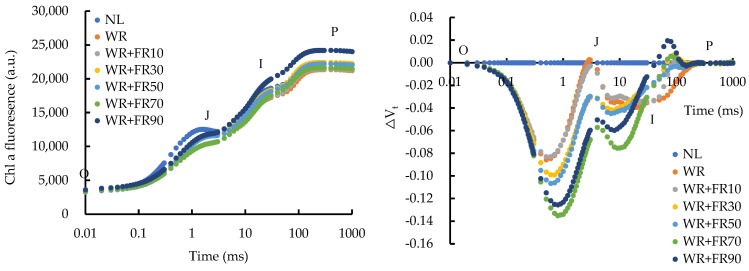
Induction curve and curve of ΔV_t_ of chlorophyll a fluorescence in lettuce leaves under different treatments, including white plus red LEDs with FR photon flux density at 0, 10, 30, 50, 70, and 90 µmol m^−2^ s^−1^ (WR, WR + FR10, WR + FR30, WR + FR50, WR + FR70, and WR + FR90, respectively), and lettuce grown with natural light only was marked as NL (three replicates).

Supplementary light significantly reduced the changes in the relative variable fluorescence (ΔV_t_) value of lettuce leaves compared to plants under natural light (NL), especially at the J and I steps (Figure 1). Specifically, the magnitude of the reduced amplitude of ΔV_t_ in lettuce leaves increased with increasing FR light intensity from 0 to 70 μmol m^−2^ s^−1^, while treatment WR + FR90 reduced the ΔV_t_ value by 12% and 59% compared to plants under treatment WR + FR70 at the J and I steps, respectively. From I to P, FR light addition with the highest intensity of 90 μmol m^−2^ s^−1^ increased the ΔV_t_ value significantly, whereas other treatments decreased the ΔV_t_ value in lettuce leaves.

**Figure 2:**
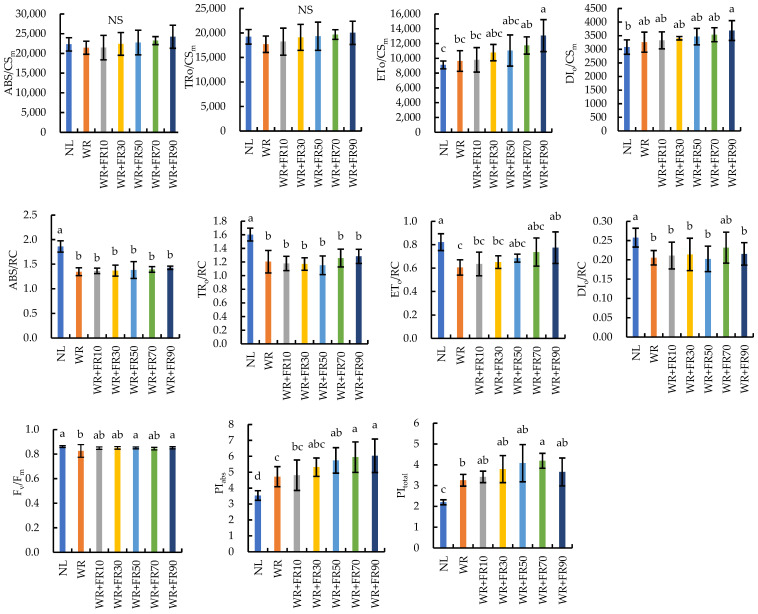
The absorption of flux per cross section (ABS/CS_m_), trapped energy flux per cross section (TR_o_/CS_m_), electron transport flux per cross section (ET_o_/CS_m_), dissipation of energy per cross section (DI_o_/CS_m_), absorption flux per reaction center (ABS/RC), trapped energy flux per reaction center (TR_o_/RC), electron transport flux per reaction center (ET_o_/RC), dissipation of energy per reaction center (DI_o_/RC), the maximum photochemical quantum yield (F_v_/F_m_), performance index based on the absorbed light energy (PI_abs_), and performance index (PI_total_) of lettuce leaves under different treatments. The light treatments included white plus red LEDs with FR photon flux density at 0, 10, 30, 50, 70, and 90 µmol m^−2^ s^−1^ (WR, WR + FR10, WR + FR30, WR + FR50, WR + FR70, and WR + FR90, respectively), and lettuce grown with natural light only was marked as NL. Means followed by the same lowercase letters and NS are not significantly different for each measured parameter, according to the least-significant difference test (*p* ≤ 0.05). Error bars indicate the standard deviation (three replicates).

**Figure 3:**
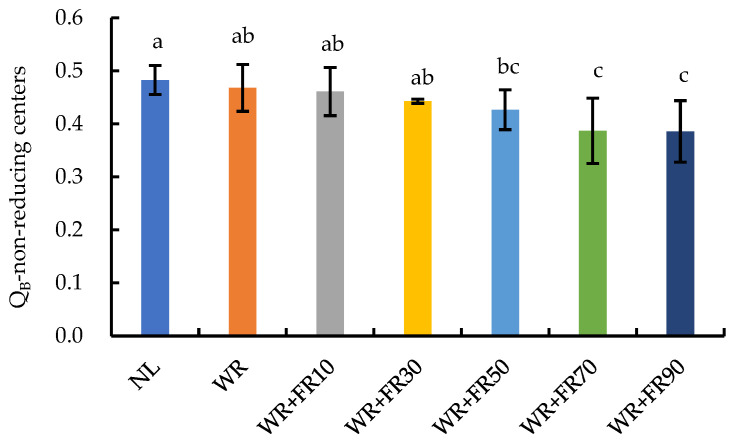
Q_B_-non-reducing centers of greenhouse-grown lettuce under different treatments, including white plus red LEDs with FR photon flux density at 0, 10, 30, 50, 70, and 90 µmol m^−2^ s^−1^ (WR, WR + FR10, WR + FR30, WR + FR50, WR + FR70, and WR + FR90, respectively), and lettuce grown with natural light only was marked as NL. Means followed by the same lowercase letters are not significantly different, according to the least-significant difference test (*p* ≤ 0.05). Error bars indicate the standard deviation (three replicates).

The chlorophyll fluorescence characteristic parameters of lettuce leaves are presented in Figure 2. Results indicated that supplementary light had no influence on the F_v_/F_m_ of lettuce plants in comparison to plants under NL, while supplementary light reduced the absorption flux per reaction center (ABS/RC), trapped energy flux per reaction center (TR_o_/RC), electron transport flux per reaction center (ET_o_/RC), and DI_o_/RC in lettuce. Conversely, the electron transport flux and dissipation of energy per cross section (ET_o_/CS_m_, DI_o_/CS_m_) of lettuce plants were increased by supplementary light treatments, which increased with increasing FR light intensity. The PI_abs_ of lettuce plants showed a similar trend, which was 41%, 41%, 38%, 33%, 26%, and 25% lower under NL than in plants under treatments WR + FR90, WR + FR70, WR + FR50, WR + FR30, WR + FR10, and WR, respectively. The PI_total_ of lettuce plants was 10% and 13% lower under treatment WR + FR90 compared to plants under treatments WR + FR70 and WR + FR50, respectively. In general, the Q_B_-non-reducing centers (Q_B_-NRC) of lettuce decreased with the increase in FR photon flux density (Figure 3). The value of Q_B_-NRC in lettuce leaves was the lowest in plants under treatments WR + FR90, WR + FR70, and WR + FR50.

### 2.2. Photosynthetic Characteristics and Chlorophyll Content of Greenhouse-Grown Lettuce

The maximum net photosynthetic rate (P_n max_) of lettuce leaves was the lowest in plants under NL and showed no difference among supplementary light treatments with different FR light intensity except plants under treatment WR + FR90 (Table 1). The dark respiration rate (R_d_) of lettuce leaves was the highest under treatments WR + FR30 and WR + FR50 and showed no difference among other treatments. The light compensation point (L_c_) of lettuce leaves was the highest in treatments WR + FR30 and WR + FR50, followed by treatments WR + FR10, WR + FR70, and WR + FR90, and the lowest in treatments WR and NL.

According to the light response curve, supplementary light showed a significantly positive effect on the P_n_ of lettuce leaves (Supplementary Figure S1). Specifically, P_n_ of lettuce leaves were in the order as below: WR + FR30 and WR + FR50 > WR > WR + FR10 > WR + FR70 > WR + FR90 > NL. When the light intensity was below 1000 μmol m^−2^ s^−1^, P_n_ in treatment WR + FR50 was lower than plants in treatment WR + FR30 and gradually increased over the one in WR + FR30 when the light intensity exceeded 1000 μmol·m^−2^·s^−1^.

**Figure 4:**
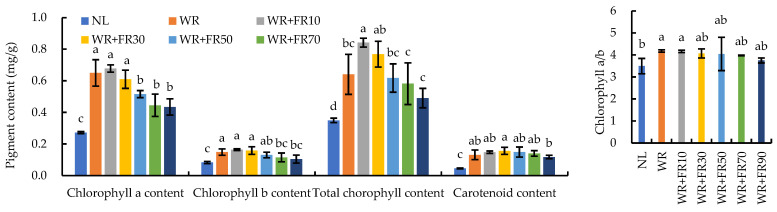
Photosynthetic pigment contents and chlorophyll a/b ratio of greenhouse-grown lettuce grown under different treatments, including white plus red LEDs with FR photon flux density at 0, 10, 30, 50, 70, and 90 µmol m^−2^ s^−1^ (WR, WR + FR10, WR + FR30, WR + FR50, WR + FR70, and WR + FR90, respectively), and lettuce grown with natural light only was marked as NL. Means followed by the same lowercase letters are not significantly different for each measured parameter, according to the least-significant difference test (*p* ≤ 0.05). Error bars indicate the standard deviation (three replicates).

Supplementary light had a significant impact on chlorophyll content in greenhouse-grown lettuce (Figure 4). The total chlorophyll content was the highest in lettuce plants under treatments WR + FR10 and WR + FR30, followed by WR, WR + FR50, WR + FR70, and WR + FR90, and the lowest under NL, which was attributed to increased chlorophyll a and b contents. The total chlorophyll content in lettuce plants was 97%, 140%, 120%, 77%, 66%, and 40% greater under treatments WR, WR + FR10, WR + FR30, WR + FR50, WR + FR70, and WR + FR90 in comparison to plants under NL, respectively. The chlorophyll a/b of lettuce was the lowest under NL and showed no difference among the six supplemental lighting treatments. The chlorophyll a/b in lettuce plants was 20%, 19%, 17%, 16%, 14%, and 7% greater under treatments WR, WR + FR10, WR + FR30, WR + FR50, WR + FR70, and WR + FR90 in comparison to plants under NL, respectively. Carotenoid content in lettuce plants was increased by 160%, 200%, 220%, 200%, 180%, and 140% under treatments WR, WR + FR10, WR + FR30, WR + FR50, WR + FR70, and WR + FR90 compared to plants under NL, respectively.

### 2.3. Growth and Biomass Accumulation of Greenhouse-Grown Lettuce

Leaf number, leaf width, and leaf area of lettuce plants showed a similar trend under supplementary light treatments, which was the highest under treatment WR + FR50, the lowest under NL, and showed no difference among other treatments (Table 2). Leaf number, leaf width, and leaf area of lettuce plants were 44%, 37%, and 84% higher under treatment WR + FR50 than those in plants under NL, respectively. By contrast, supplementary light significantly decreased the leaf length and length-width ratio of lettuce leaves. The specific leaf area of lettuce plants was decreased by supplementary light treatments, no matter with or without FR light addition.

**Figure 5:**
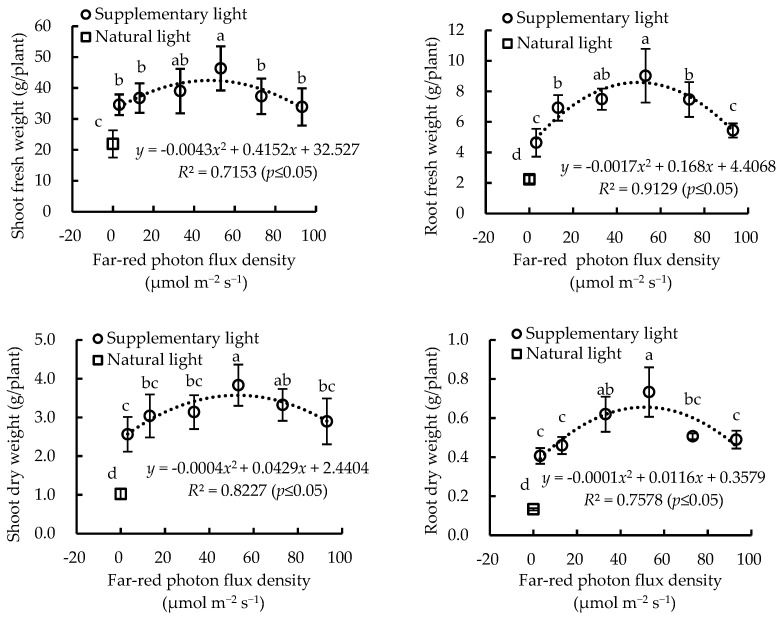
Regressions between FR photon flux density and biomass accumulation of greenhouse-grown lettuce grown under different treatments, including white plus red LEDs with FR photon flux density at 0, 10, 30, 50, 70, and 90 µmol m^−2^ s^−1^ (WR, WR + FR10, WR + FR30, WR + FR50, WR + FR70, and WR + FR90, respectively), and lettuce grown with natural light only were marked as NL. Error bars indicate the standard deviation (three replicates).

**Figure 6:**
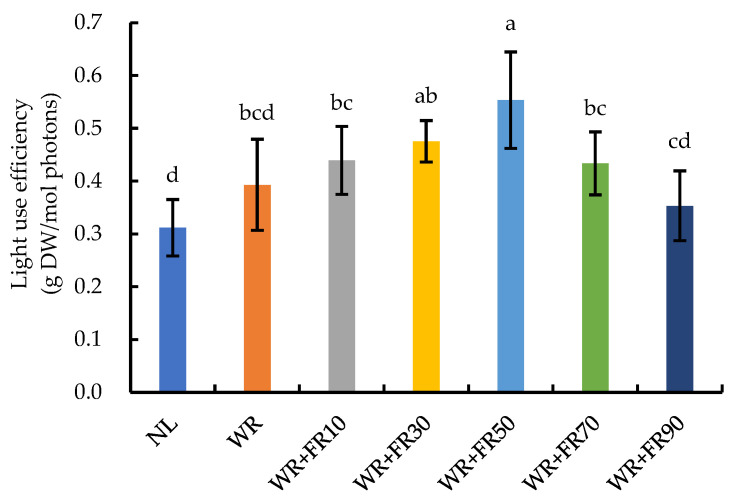
Light use efficiency of greenhouse-grown lettuce under different treatments, including white plus red LEDs with FR photon flux density at 0, 10, 30, 50, 70, and 90 µmol m^−2^ s^−1^ (WR, WR + FR10, WR + FR30, WR + FR50, WR + FR70, and WR + FR90, respectively), and lettuce grown with natural light only was marked as NL. Error bars indicate the standard deviation (three replicates).

**Figure 7:**
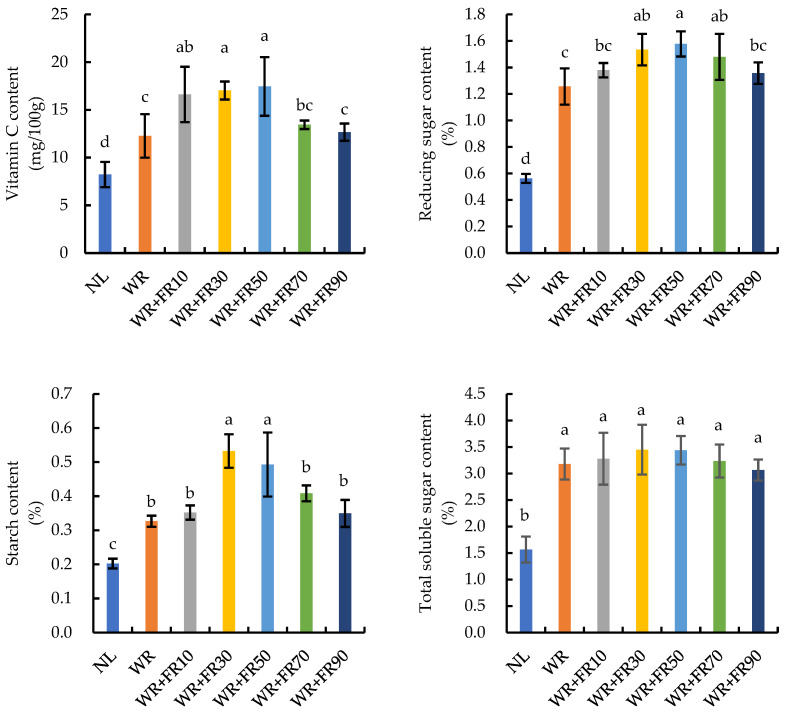
Vitamin C, reducing sugar, total soluble sugar, and starch content of greenhouse-grown lettuce under different treatments, including white plus red LEDs with FR photon flux density at 0, 10, 30, 50, 70, and 90 µmol m^−2^ s^−1^ (WR, WR + FR10, WR + FR30, WR + FR50, WR + FR70, and WR + FR90, respectively), and lettuce grown with natural light only was marked as NL. Means followed by the same lowercase letters are not significantly different, according to the least-significant difference test (*p* ≤ 0.05). Error bars indicate the standard deviation (three replicates).

Supplementary light increased the fresh and dry weights, as well as the light use efficiency (LUE) of lettuce plants, compared to those grown with only sunlight (Figure 5 and Figure 6). Shoot and root fresh/dry weight of lettuce plants increased first and decreased subsequently, following a quadratic function with increasing FR photon flux density (Figure 6). Lettuce plants grown under 50 μmol m^−2^ s^−1^ FR light addition led to greater biomass accumulation than those grown with 10, 30, 70, and 90 μmol m^−2^ s^−1^ FR light addition. Shoot fresh weight, root fresh weight, shoot dry weight, and root dry weight of lettuce plants were increased by 111%, 303%, 275%, and 462%, respectively, under treatment WR + FR50 compared with those grown under NL. Meanwhile, the LUE of lettuce plants was remarkably prompted by increasing supplementary FR light intensity from 0 to 50 μmol m^−2^ s^−1^, and the improvement effect was reduced with FR light intensity over 50 μmol m^−2^ s^−1^ (Figure 7).

### 2.4. Vitamin C, Sugar, and Starch Contents of Greenhouse-Grown Lettuce

Supplementary light caused a significant rise in the contents of vitamin C, reducing sugar, total soluble sugar, and starch in lettuce plants compared to plants under NL (Figure 7). The vitamin C content in lettuce plants was the highest under treatments WR + FR10, WR + FR30, and WR + FR50, followed by treatments WR, WR + FR70, and WR + FR90, and the lowest under NL. The vitamin C content in lettuce plants under NL was 33%, 52%, 52%, 53%, 39%, and 35% lower than in plants under treatments WR, WR + FR10, WR + FR30, WR + FR50, WR + FR70, and WR + FR90, respectively. The reducing sugar content and starch content in lettuce showed a similar trend, whereas the total soluble sugar content was lower in plants under NL and showed no differences among the six supplementary light treatments.

## 3. Discussion

Light energy that is absorbed by chlorophyll in photosynthetic systems can undergo three fates: photochemistry, heat dissipation, and fluorescence emission [24]. Thus, chlorophyll fluorescence is commonly used as a tool to provide precise and objective information regarding photochemical efficiency and non-photochemical de-excitation involved in the conversion of light energy under different conditions [25,26]. In the present study, as FR light intensity increased, the value of ΔV_I_ and ΔV_J_ of lettuce leaves decreased gradually except for treatment WR + FR90 (Figure 1), indicating less accumulation of PSII acceptor quencher (QA) in the photosynthetic electron transport chain under FR light addition, which promoted electron transport capacity of PSII and CO_2_ assimilation [24,27,28]. In the case of the increased chlorophyll fluorescence value of lettuce leaves at the O-I-P point under treatment WR + FR90 (Figure 1), a higher FR photon intensity, or FR proportion, reduced the electron transport capacity of PSII and the proportion of absorbed light energy used for photochemical reactions [27,29]. Furthermore, supplementary light increased the number of active reaction centers as well as the electron transfer efficiency per cross section, which was explained by the decreased ABS/RC and TR_o_/RC values with similar ABS/CS_m_ and TR_o_/CS_m_ values (Figure 2). This was consistent with the decreased value of Q_B_-NRC with increasing FR photon flux density (Figure 3), suggesting an increase in the number of PSII photochemically active reaction centers [30]. The smaller reduction of ET_o_/RC compared to ABS/RC and TR_o_/RC suggested that FR light addition promoted electron transfer efficiency per active reaction center, and its lifting effect increased with FR photon flux density (Figure 2). The photosynthetic performance index PI_abs_ is a performance index based on the absorbed light energy, which mainly reflects the efficiency of the reaction center of PSII, while PI_total_ can further reflect the ability of electron transport between PSII and PSI and the related properties of PSI [31]. The greater increase of PI_total_ than PI_abs_ in lettuce leaves under supplementary light in comparison to NL suggested higher electron transfer efficiency within the photosynthetic electron transport chain, which is positively correlated with FR light intensity except for the FR photon flux density of 90 μmol m^−2^ s^−1^, due to an overexcitation of PSI to PSII [6,32]. Taken together, enhanced cyclic electron transport and photochemical efficiency by FR light addition exhibited a saturation response to the dose of FR light, and a high FR light intensity of 90 μmol·m^−2^·s^−1^ inhibited plant photochemistry efficiency, which is due to an unbalanced excitation between the two photosystems [6,33].

Photosynthesis is the basis for material accumulation, and recent research indicated that adding FR light to PAR wavelengths in both fully controlled environments with artificial lighting and semi-closed facilities with sunlight has a positive influence on leaf and canopy photosynthesis in lettuce, sunflower (*Helianthus annuus* L.), corn (*Zea mays* L.), burdock (*Arctium minus* Bernh.), and Norway maple (*Acer platanoides* L.) plants [7,34]. The P_n max_ represents the photosynthetic potential of a plant, and the larger the value, the more photosynthetic products are synthesized by plants under the same light conditions [27,35]. In the present study, the promotion of P_n max_, R_d_, and L_c_ in lettuce leaves by supplementary light indicated an improved plant’s ability to utilize strong light. Although P_n max_ showed no significant difference among different FR light intensities, there was a trend that P_n max_ increased with increasing FR photon intensity from 0 to 50 μmol m^−2^ s^−1^ and decreased gradually over 50 μmol m^−2^ s^−1^ (Table 1, Figure 3). This agreed with previous studies conducted in IVF that at a given level of PAR (PPFD around 200 μmol m^−2^ s^−1^ provided by a combination of red and blue light, photoperiod of 16 h), only a certain amount of FR photons (50 μmol m^−2^ s^−1^) increased the P_n_ and φPSII in lettuce plants through better balanced excitation of the two photosystems, and increasing FR photons showed no further improvement or even deterioration [6,8]. The consistent conclusion between our experiment in greenhouses and previous studies in IVFs might be contributed to the similar lighting environment (PPFD around 200 μmol·m^−2^·s^−1^ with FR photon flux density about 30 to 50 μmol·m^−2^·s^−1^) among different experiments [6,8,9]. In the present study, the lighting environment in the greenhouse during FR light application was mainly provided by supplementary artificial lighting, which was applied for a photoperiod of 12 h d^−1^ (04:00–10:00 and 14:00–20:00) from late Nov. to Dec. (sunrise around 07:00 and sunset around 16:40), during which the sunlight intensity in the greenhouse (including PAR and FR wavelength) is mostly lower than 50 μmol m^−2^ s^−1^.

FR light addition adjusts light-harvesting structure to increase light harvesting ability and carbon assimilation of plants, which was attributed to plant shade-avoidance or shade-tolerance responses [36,37]. Shade-avoidance responses often manifest as greater hypocotyl/stem/petiole elongation and reduced branching under shade, while shade-tolerance responses show leaf expansion with reduced leaf thickness [38]. A significant shade-tolerance response to leaf expansion by supplementary FR light was observed in this study, which was mainly caused by increased leaf number and/or leaf width instead of leaf length (Table 2). Similar effects by FR light addition as photoperiodic lighting in lettuce and other plant species such as geranium (*Pelargonium × hortorum*), snapdragon (*Antirrhinum majus*), radish (*Raphanus sativus*), and kale (*Brassica napus*) were observed in previous studies in IVF, which facilitated better light interception and led to higher plant biomass accumulation [13,37,38,39,40]. Jin et al. investigated the underlying components of FR effects (52 μmol m^−2^ s^−1^ FR light as photoperiodic lighting added to R&B light of 218 μmol m^−2^ s^−1^) on lettuce growth, and results indicated that FR increased plant dry weight by 46–77%, which was mainly due to a higher canopy-intercepted PPFD caused by increased leaf area, and to a smaller extent (8–23%) by higher intercepted LUE [9]. As Legendre and van Iersel stated, each 1 m^2^ increase in lettuce leaf area was associated with a 59 mol increase in incident light after 25 days of growth [10]. Thus, this can be a self-reinforcing process: plants under FR light treatment developed a larger leaf area to absorb more light, grow faster, and produce additional leaf area faster than plants under treatment without FR light [41,42]. Specific leaf area has often been found to increase with additional FR light at a shorter wavelength [13,43], but not in the current research or study by Jin et al., which may be due to varied FR effects on different lettuce species and cultivars [9,44]. In this study, supplementary light was used during 04:00–10:00 and 14:00–20:00 (sunrise around 07:00 and sunset around 16:40 during the experiment), which meant the extended photoperiod was applied and the natural photoperiod was extended by approximately 3 h in the beginning and at the end of the natural daylight period, respectively, leading to the bigger leaf area and higher dry weight of lettuce (Table 2 and Figure 5). Zou et al. reported that adding FR to red plus blue light either during the day (16 h) or end-of-day (EOD, 1 h) improved biomass production of lettuce compared with those grown without FR; however, additional FR light at EOD led to lower leaf area and lower dry weight of lettuce than those grown under FR during the day [13]. The differences may be related to the supplementary light duration. In addition, with the same light intensity and duration (50 μmol m^−2^ s^−1^ for 1 h), applying FR at the EOD without PAR resulted in a bigger leaf area and higher biomass of lettuce compared with those FR lights applied at the EOD with PAR, indicating that FR light, with the appropriate supplementary time, proved to be more effective in enhancing growth and improving radiation use efficiency [6].

Notably, supplementary FR light to short wavelengths means a decreased ratio of R:FR, which is linked to many light effects on plant morphogenesis, growth, and metabolic processes mediated by phytochromes [45,46,47]. Phytochromes, which exist in two photo-interconvertible isomeric forms: a red-light-absorbing form (Pr, λmax = 660 nm, biologically inactive form) and a FR-light-absorbing form (Pfr, λmax = 730 nm, biologically active form), enable plants to sense and adapt to the light environment, thereby improving energy efficiency, growth patterns, and resilience to abiotic stresses [37,45,47]. For example, a lower R:FR condition improved tomato salinity stress tolerance, and phytochrome B1 plays an important role in this process [47]. In the present study, the R:FR ratio provided by treatments WR, WR + FR10, WR + FR30, WR + FR50, WR + FR70, and WR + FR90 was 15.72, 3.75, 1.49, 0.93, 0.67, and 0.53, respectively. It clearly indicated that a decreased R:FR ratio (no lower than 0.93) boosted lettuce growth and biomass accumulation through both morphological and photochemical effects. Interestingly, this R:FR ratio of 0.93 (WR + FR50) is similar to the R:FR ratio of sunlight at noon (around 1.0 to 1.3), which is proven suitable for plant growth as it supports optimal phytochrome activity, efficient photosynthesis, and favorable morphological traits [48]. Further, this was consistent with the suggestion by Zhen and Bugbee that a FR proportion of 35% (the R:FR ratio was not provided) increased lettuce photosynthetic rates [7]. However, Kusuma and Bugbee found a positive effect of FR on leaf area and dry mass accumulation at high PPFD but a negative effect on these growth parameters at lower PPFD, suggesting interactive effects of PPFD and FR light addition on lettuce growth, which have previously been described for other crops [11,29]. Furthermore, Zou et al. stated that the acclimation process of plant morphology triggered by FR addition made a major contribution to the yield improvement of lettuce in IVF since FR light decreased the plant’s photosynthetic capacity in the long term due to a lower chlorophyll and total nitrogen content, along with decreased leaf light absorption [13]. The decreased chlorophyll content by FR light addition was largely reported in previous literature [15,39,40,43], while in the present study, this reduction effect on chlorophyll contents was only observed with FR photon flux density over 50 μmol·m^−2^·s^−1^ (Figure 4). This might be the reason for the inconsistency of the optimal FR photon flux density for plant photochemical efficiency and biomass accumulation, which was 70 μmol m^−2^ s^−1^ and 30~50 μmol m^−2^ s^−1^, respectively, since high FR photon flux density over 50 μmol m^−2^ s^−1^ significantly decreased leaf chlorophyll content, thus leaf light absorption and LUE. Taken together, the varied background light intensity, different spectral compositions, and proportion of FR photons in total PPFD, as well as lettuce species and cultivars, all affect plant responses to supplementary FR light. According to the results of current research, in the case of greenhouse-grown lettuce produce with commonly used PPFD of 200 μmol·m^−2^·s^−1^, FR light intensity of 30~50 μmol m^−2^ s^−1^ or a FR proportion of 15~25% was suggested.

It has been widely reported that FR light addition or a low R:FR ratio would increase plant carbohydrate content, such as starch and sucrose content, mainly due to improved photochemical efficiency [8,12,49]. The changes in trends of vitamin C, reducing sugar content, and starch content were similar to the P_n max_, light use efficiency, and total chlorophyll content of lettuce in this study (Figure 7), indicating that improved photosynthetic capacity and relative CO_2_ assimilation by FR light addition possibly contributed to increased carbohydrate contents. Further, high levels of carbohydrates improve the sweetness and crispness of lettuce, which may enhance plant sensory quality and shelf life after harvest [50,51]. However, a decreased content of carotenoids and total phenolics under FR light addition was also reported in previous studies [39,52,53]. This reduction might cause crops to be more susceptible to pests and pathogens since these phytochemicals contribute to plants’ defense against insects, microbial pathogens, and fungi [43,52]. This might be a result of plants investing resources in the most efficient way into plant expansion growth instead of phytochemical synthesis [38,54]. Further experiments are required to clarify how FR light influences the synthesis of these secondary metabolites.

## 4. Materials and Methods

### 4.1. Plant Materials

Lettuce ‘Dasusheng’ (*Lactuca sativa* L.) was selected as the experimental material. All lettuce seeds were grown in 72-cell plug trays filled with mixed peat (The Pindstrup Group, Kongersle, Denmark), vermiculite (Shandong Lige Technology Co., Ltd., Jinan, China), and perlite (Shandong Lige Technology Co., Ltd., Jinan, China) (3:1:1, *v*/*v*/*v*) in a commercial greenhouse. Lettuce seedlings with four true leaves were selected for uniformity and transplanted into 1.8 L plastic pots (diameter, 16.8 cm; depth, 14 cm) one day before treatment. The plastic pots were filled with mixed peat, vermiculite, and perlite (3:1:1, *v*/*v*/*v*) and kept in a Venlo-type greenhouse at Qingdao Agricultural University (36°19′ N, 120°23′ E), Qingdao, China. The experiment was conducted for 35 days (from 21 November to 25 December 2023), and the air temperature was maintained at 22 ± 3 °C/18 ± 3 °C (day/night), while the relative humidity was maintained at 60~70%. Lettuce was cultivated using Hoagland’s nutrient solution according to a previously reported method [55].

### 4.2. Light Treatment Design

**Figure 8:**
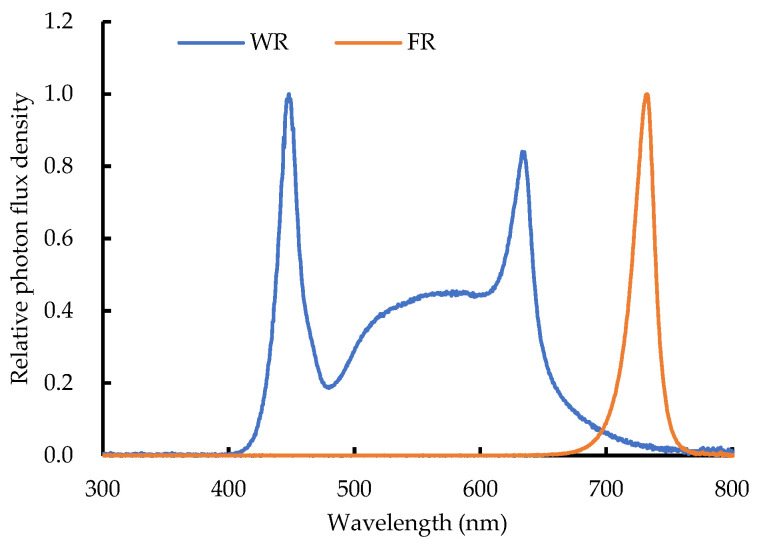
Spectral distribution of supplementary white-red (WR) light-emitting diodes (LEDs) and far-red LEDs (FR).

A randomized complete block design was implemented to evaluate the effect of supplementary FR light at shorter wavelengths on greenhouse-grown lettuce. According to our previous study, white plus red LEDs (WR, color temperature of 6500 K, provided by Zhongshan Aier Lighting Technology Co., Ltd., Zhongshan, China) with a DLI of 6.52 mol m^−2^ d^−1^ were suitable for lettuce growth [55]. Therefore, in this experiment, six supplemental light treatments with different FR light intensities: WR LEDs without FR light addition and WR LEDs with FR (Xiamen Lumigro Technology Co., Ltd., Xiamen, China) photon flux density at 10, 30, 50, 70, and 90 µmol m^−2^ s^−1^ (WR + FR10, WR + FR30, WR + FR50, WR + FR70, and WR + FR90, respectively) were implemented. Lettuce grown with only natural light was marked as NL. The PPFD provided by WR LEDs was set at 151 µmol m^−2^ s^−1^, and the supplementary lighting was carried out in two time slots, 04:00–10:00 and 14:00–20:00 (sunrise around 07:00 and sunset around 16:40), during which the sunlight intensity in the greenhouse is mostly lower than 50 μmol m^−2^ s^−1^. The average DLI of solar light inside the greenhouse was 5.0 mol m^−2^ d^−1^, while the DLI of supplemental light treatments was 6.52 mol m^−2^ d^−1^. For treatment WR, WR + FR10, WR + FR30, WR + FR50, WR + FR70, and WR + FR90, the R:FR ratio provided by LEDs was 15.72, 3.75, 1.49, 0.93, 0.67, and 0.53, respectively. The spectral distribution of each treatment was measured with a spectrometer (AvaSpec-ULS2048-USB2, Avantes Inc., Apeldoorn, The Netherlands) above the plant canopy (Table 3 and Figure 8). The peak wavelengths of the WR LEDs and FR light lamps are 446 nm (B), 633 nm (R), and 730 nm (FR), respectively. In this experiment, each treatment was replicated in three blocks. For each block, 20 lettuce plants were cultivated with a planting density of 25 plants m^−2^. At harvest, three uniform plants were randomly selected in each block for measurement.

### 4.3. Growth Measurement

#### 4.3.1. Photosynthetic Characteristics, Pigments, and Chlorophyll Fluorescence

A light response curve was determined by a portable photosynthesis system (Li-6400XT, LI-COR Corporation, Lincoln, NE, USA) for lettuce leaves. Measurement was conducted in clear weather, and PPFD inside the leaf chamber was set to eight gradients of 2000, 1500, 1000, 500, 200, 100, 50, and 0 µmol m^−2^ s^−1^, respectively. Leaf temperature, gas flow rate, and the CO_2_ concentration inside the leaf chamber were set at 22 °C, 500 µmol s^−1^, and 400 µmol mol^−1^, respectively. The maximum net photosynthetic rate (P_n max_), dark respiration rate (R_d_), and light compensation point (L_c_) were calculated based on the light response curve according to the Ye Model [56].

Chlorophyll fluorescence parameters of lettuce were measured at harvest using a pocket Plant Efficiency Analyzer chlorophyll fluorimeter (PEA, Hansatech Instruments Ltd., Norfolk, UK). Leaves were darkly adapted for at least 30 min prior to a rapid chlorophyll -a fluorescence induction kinetic curve (OJIP curve) measurement. Calculation of chlorophyll a fluorescence parameters including F_v_/F_m_, ABS/RC, TR_o_/RC, ET_o_/RC, DI_o_/RC, ET_o_/CS_m_, DI_o_/CS_m_, ABS/CS_m_ and TR_o_/CS_m_, PI_abs_, and PI_total_ were based on Li et al. [57]. The proportion of QB-NRC was calculated based on the method reported by Klinkovsky and Naus [58]. To help visualize the influence of supplementary light on chlorophyll a fluorescence transients of lettuce leaves, the value of relative variable fluorescence, V_t_ = (F_t_ − F_o_)/(F_m_ − F_o_), was calculated. Further, the changes in relative variable fluorescence (ΔV_t_) were calculated by subtracting the values of the fluorescence recorded in plants under supplementary treatments from those recorded for NL plants.

At harvest, three plants were selected for the measurement of chlorophyll content and carotenoids’ content within each block, and the third leaf from the top was selected for measurement. Fresh lettuce leaves were cut into small pieces and then extracted in 80% acetone (*v*/*v*) for 24 h. The absorbance of the extracted supernatants was measured at 663, 645, and 470 nm, respectively, using a UV-VIS spectrophotometer (1810, Shanghai Yoke Instrument Co., Ltd., Shanghai, China). The concentrations of chlorophyll a, chlorophyll b, and carotenoids were calculated according to Lichtenthaler and Wellburn [59] and expressed as mg g^−1^ (fresh weight). The total chlorophyll content and ratio of chlorophyll a/b were calculated accordingly.

#### 4.3.2. Plant Morphology and Growth Characteristics

Leaf number, leaf length and width of the maximum leaf blade, and shoot and root fresh weight (using an electronic analytical balance, JY20002, Shanghai Hengping Instrument Co., Ltd., Shanghai, China) were recorded at harvest (35 days after treatment). Leaf area was measured by a leaf area scanner (Yaxin-1241, Beijing Yaxin Liyi Technology Co., Ltd., Beijing, China). Fresh leaves and roots were dried in an oven at 105 °C for 3 h, and then dried at 80 °C for 72 h to measure the shoot and root dry weight.

#### 4.3.3. Vitamin C, Reducing Sugar, Soluble Sugar, and Starch Content

At harvest, fresh lettuce leaves (the third leaf from the top) were selected for the measurement of vitamin C using the 2, 6-dichlorophenol indophenol titration method according to Shyamala and Jamuna [60] and expressed as mg 100g^−1^ (fresh weight). Dried leaf samples were selected for the measurement of soluble sugar content and reducing sugar content using the anthronesulfuric acid colorimetry method and the 5-dinitrosalicylic acid colorimetric method, according to Song et al. [61] and Zhan et al. [62], respectively. The starch content of dried leaf samples was measured according to Takahashi et al. [63]. The measurement of soluble sugar, reducing sugar, and starch contents was done using a UV-VIS spectrophotometer (1810, Shanghai Yoke Instrument Co., Ltd., Shanghai, China) and was expressed as a percentage (dry weight).

### 4.4. Statistical Analysis

Data collected from the three blocks for each treatment were analyzed, and one-way analysis of variance (ANOVA) and the least significant difference (LSD) test (*p* ≤ 0.05) were carried out using SPSS 26.0 software (IBM, Inc., Chicago, IL, USA) to reveal the difference among groups. The data were expressed as the means ± standard deviations (SD). Regressions between treatments and the morphological characteristics of lettuce were performed using Microsoft Excel 2021 software.

## 5. Conclusions

Our results demonstrated that the addition of FR light to the PAR wavelength differentially influenced the photochemical efficiency of PSII as well as the electron transfer efficiency between PSII and PSI, which was positively correlated with the FR photon flux density from 10 to 70 μmol m^−2^ s^−1^. However, shoot fresh weight of lettuce plants was increased by 26%, 19%, 24%, and 37%, respectively, under treatment WR + FR50 compared with those grown under 10, 30, 70, and 90 μmol m^−2^ s^−1^ FR light addition. A similar trend was observed: the contents of vitamin C and carbohydrates in lettuce were enriched the most under FR addition of 30~50 μmol m^−2^ s^−1^. This is probably because the FR photon flux density of 70~90 μmol m^−2^ s^−1^ significantly decreased leaf total chlorophyll content by 7~21% compared to treatment WR + FR50, thus decreasing leaf light absorption and LUE accordingly. Therefore, with commonly used growing light of PPFD around 200 μmol m^−2^ s^−1^, addition of FR light as photoperiodic light around 30~50 μmol m^−2^ s^−1^ was suggested to enhance the photochemistry efficiency, biomass accumulation, and carbohydrate contents in greenhouse-grown lettuce.

## Figures and Tables

**Table 1:** The maximum net photosynthetic rate (P_n max_), dark respiration rate (R_d_), and light compensation point (L_c_) of greenhouse-grown lettuce leaves under different treatments, including white plus red LEDs with FR photon flux density at 0, 10, 30, 50, 70, and 90 µmol m^−2^ s^−1^ (WR, WR + FR10, WR + FR30, WR + FR50, WR + FR70, and WR + FR90, respectively), and lettuce grown with natural light only was marked as NL. Means followed by the same lowercase letters within the column are not significantly different, according to the least-significant difference test (*p* ≤ 0.05). Errors indicate the standard deviation (three replicates).

Treatments	P_n max_ (μmol m^−2^ s^−1^)		R_d_ (μmol m^−2^ s^−1^)		L_c_ (μmol m^−2^ s^−1^)	
NL	6.5 ± 1.1	c	0.84 ± 0.09	b	12.6 ± 2.3	c
WR	10.2 ± 0.9	ab	0.88 ± 0.16	b	12.7 ± 1.8	c
WR + FR10	10.7 ± 2.0	a	1.01 ± 0.05	b	17.2 ± 3.1	b
WR + FR30	11.6 ± 2.6	a	1.73 ± 0.34	a	20.1 ± 4.5	ab
WR + FR50	11.7 ± 1.5	a	1.53 ± 0.14	a	21.3 ± 1.3	a
WR + FR70	10.5 ± 0.1	ab	1.09 ± 0.14	b	16.5 ± 1.7	bc
WR + FR90	8.0 ± 1.3	bc	1.01 ± 0.18	b	16.1 ± 2.9	bc

**Table 2:** Leaf morphological traits of greenhouse-grown lettuce under different treatments, including white plus red LEDs with FR photon flux density at 0, 10, 30, 50, 70, and 90 µmol m^−2^ s^−1^ (WR, WR + FR10, WR + FR30, WR + FR50, WR + FR70, and WR + FR90, respectively), and lettuce grown with natural light only was marked as NL. The means followed by the same lowercase letters within the column are not significantly different, according to the least-significant difference test (*p* ≤ 0.05). Errors indicate the standard deviation (three replicates).

Treatments	Leaf Number		Leaf Length (cm)		Leaf Width (cm)		Length-Width Ratio		Leaf Area (cm^2^)	Specific Leaf Area (cm^2^ g^−1^)		
NL	9.0 ± 0.7	c	16.8 ± 0.8	a	12.0 ± 0.6	c	1.40 ± 0.09	a	561 ± 76	d	1121 ± 97	a
WR	11.0 ± 1.0	b	14.2 ± 0.2	b	14.8 ± 1.4	b	0.97 ± 0.09	b	678 ± 131	cd	506 ± 36	b
WR + FR10	11.4 ± 0.5	b	14.0 ± 0.6	bc	15.0 ± 1.1	b	0.94 ± 0.09	bc	745 ± 40	c	469 ± 23	b
WR + FR30	11.8 ± 0.8	b	12.8 ± 0.4	d	15.2 ± 0.6	ab	0.85 ± 0.06	cd	945 ± 73	ab	445 ± 33	b
WR + FR50	13.0 ± 1.2	a	12.6 ± 1.1	d	16.4 ± 0.5	a	0.77 ± 0.07	d	1031 ± 138	a	448 ± 45	b
WR + FR70	11.8 ± 0.8	b	13.2 ± 0.8	cd	14.8 ± 1.3	b	0.90 ± 0.11	bc	811 ± 89	bc	500 ± 17	b
WR + FR90	11.4 ± 0.5	b	13.3 ± 0.8	bcd	14.2 ± 1.2	b	0.95 ± 0.07	bc	730 ± 37	c	507 ± 39	b

**Table 3:** The photosynthetic photon flux density (PPFD, 400–700 nm), ratio of blue (B, 400–499 nm), green (G, 500–599 nm), and red (R, 600–699 nm) photon flux density, far-red photon flux density (FR, 700–800 nm), and R:FR ratio of different supplementary lighting treatments, including white plus red LEDs with FR photon flux density at 0, 10, 30, 50, 70, and 90 µmol m^−2^ s^−1^ (WR, WR + FR10, WR + FR30, WR + FR50, WR + FR70, and WR + FR90, respectively).

Treatments	PPFD (µmol m^−2^ s^−1^)	Ratio of B:G:R	FR Photon Flux Density (µmol m^−2^ s^−1^)	Ratio of R:FR
WR	151	1:1.36:1.14	3	15.72
WR + FR10			13	3.75
WR + FR30			33	1.49
WR + FR50			53	0.93
WR + FR70			73	0.67
WR + FR90			93	0.53

## Data Availability

Data are contained in the article. Please contact the corresponding author for any additional information.

## Supplementary Materials

The following supporting information can be downloaded at: https://www.mdpi.com/article/10.3390/plants13152169/s1, Figure S1: Photosynthetic response curves of lettuce leaves under different treatments, including white plus red LEDs with FR photon flux density at 0, 10, 30, 50, 70, and 90 µmol m^−2^ s^−1^ (WR, WR + FR10, WR + FR30, WR + FR50, WR + FR70, and WR + FR90, respectively), and lettuce grown with natural light only was marked as NL.
