# Supplementary material for: Supplementary Far-Red Light for Photosynthetic Active Radiation Differentially Influences the Photochemical Efficiency and Biomass Accumulation in Greenhouse-Grown Lettuce
Source: Plants (Basel). 2024 Aug 5;13(15):2169. doi: 10.3390/plants13152169 (PMC11314222; doi:10.3390/plants13152169)
Supplement: Supplementary file 1 [file plants-13-02169-s001.zip › plants-3107742-supplementary.pdf]

Supplementary Material

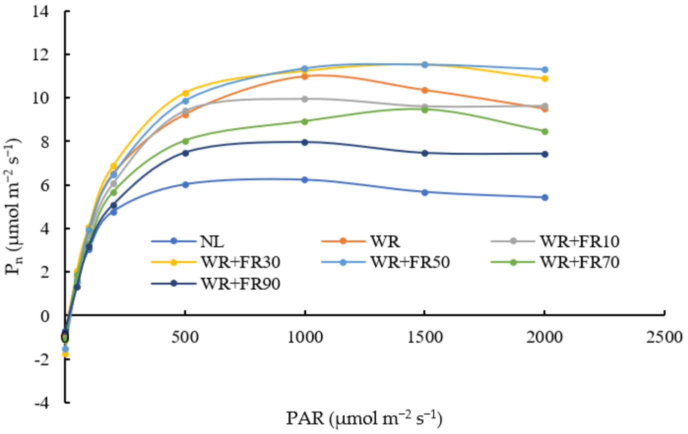

Supplementary Figure S1. Photosynthetic response curves of lettuce leaves under different treatments, including white plus red LEDs with FR photon flux density at 0, 10, 30, 50, 70, and 90 μmol m^{-2} s^{-1} (WR, WR + FR10, WR + FR30, WR + FR50, WR + FR70, and WR + FR90, respectively), and lettuce grown with natural light only was marked as NL.
